# Exploring new frontiers: a rare case of catheter ablation for persistent atrial fibrillation in a patient with cor triatriatum sinister guided by intracardiac echocardiography

**DOI:** 10.1186/s13019-024-02859-9

**Published:** 2024-06-22

**Authors:** Hengli Lai, Bo Wu, Yu Tao, Haiqiang Ding, Yanfeng Liu, Zhiyun Zhu, Xiantao Huang, Hongyan Li, Zhicheng Xu, Zhenhuan Chen, Haiwen Zhou

**Affiliations:** 1 Department of Cardiology, Jiangxi Provincial People’s Hospital, The First Affiliated Hospital of Nanchang Medical College, Nanchang, Jiangxi 330006 P. R. China; 2 Jiangxi Medical College, Nanchang University, Nanchang, Jiangxi 330006 P. R. China

**Keywords:** Cor triatriatum sinister, Persistent atrial fibrillation, Intracardiac echocardiography, Catheter ablation

## Abstract

**Background:**

Cor triatriatum sinister (CTS) is an uncommon congenital cardiac anomaly. Atrial fibrillation (AF) is commonly the initial symptom in patients with CTS, occurring in approximately 32% of the cases. The complexity of performing AF catheter ablation, particularly in cases with persistent AF, increases in patients with CTS due to its unique structural challenges.

**Case presentation:**

We report the treatment course of a 60-year-old male patient diagnosed with CTS, who underwent catheter ablation of drug-refractory, persistent AF. The complex anatomical structure of the condition made catheter ablation of AF challenging. To navigate these challenges, we performed comprehensive assessments using transthoracic echocardiography and transesophageal echocardiography, along with cardiac computed tomography angiography, prior to treatment initiation. The intricate anatomy of CTS was further clarified during the procedure via intracardiac echocardiography (ICE). Additionally, the complexity of catheter manipulation was further reduced with the aid of the VIZIGO sheath and the vein of Marshall ethanol infusion to achieve effective mitral isthmus blockage, thereby circumventing the impact of the CTS membrane.

**Conclusions:**

This case underscores the complexity and potential of advanced ablation techniques in managing cardiac arrhythmias associated with unusual cardiac anatomies. During the procedure, ICE facilitated detailed modeling of the left atrium, including the membranous structure and its openings, thus providing a clearer understanding of CTS. It is noteworthy that the membrane within the CTS may serve as a potential substrate for arrhythmias, which warrants further validation through larger sample studies.

**Supplementary Information:**

The online version contains supplementary material available at 10.1186/s13019-024-02859-9.

## Case presentation

**Fig. 1:**
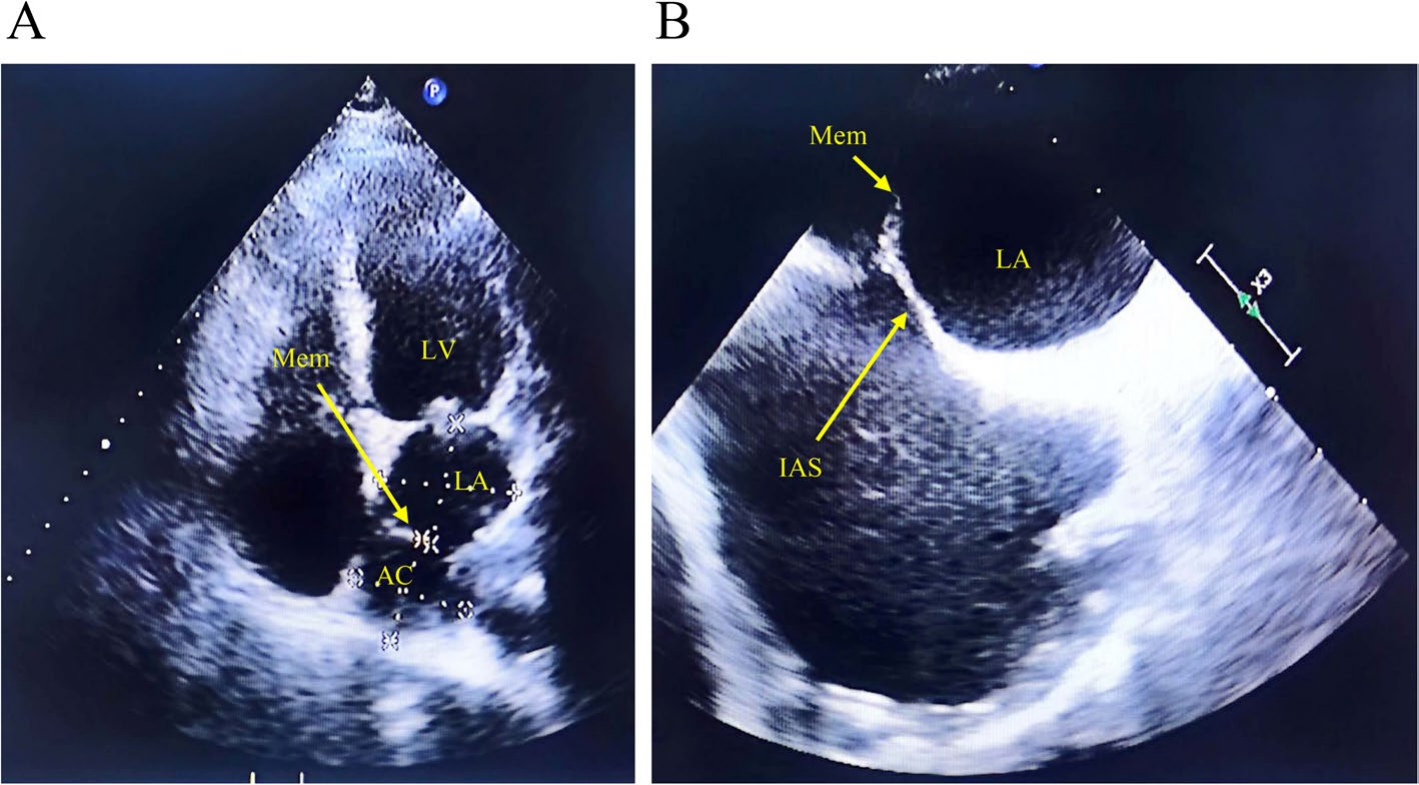
Images obtained using transthoracic and esophageal ultrasound. **A** Image obtained using transthoracic ultrasound. **B** Image obtained using transesophageal ultrasound. A fibromuscular membrane divided the left atrium into accessory and true left atrial chambers. Mem: membrane; AC: accessory chamber; LA: true left atrial chamber; LV: left ventricle; IAS: interatrial septum

A 60-year-old male patient presented with persistent palpitations and chest discomfort that was adversely affecting his exercise tolerance for over half a month despite taking oral metoprolol tartrate tablets and rivaroxaban. The patient denied any history of chronic conditions such as diabetes, coronary heart disease, or hypertension. Preoperative assessments revealed that the blood counts, electrolytes, lipid profile, hepatic and renal function, coagulation status, thyroid function, cardiac enzymes, and troponin were all within normal limits, except for a marginally elevated N-terminal pro b-type natriuretic peptideat 775.9 pg/mL. An electrocardiogram documented rapid atrial fibrillation (AF), specifically, a ventricular rate of 122 beats per minute, confirming the requirement for further medical intervention. Preoperative transthoracic echocardiography (TTE) and transesophageal echocardiography (TEE) identified a membranous structure within the left atrium, delineating an accessory and a true left atrial chamber (Fig. 1a, b). Measurements indicated the accessory left atrial chamber was 39 × 32 mm, the true left atrial chamber was 46 × 20 mm, and the communication in the membrane was 20 mm wide. Right atrial dimensions were 49 × 62 mm, and the left ventricular end-diastolic diameter was 50 mm, with an ejection fraction of 56%.

**Fig. 2:**
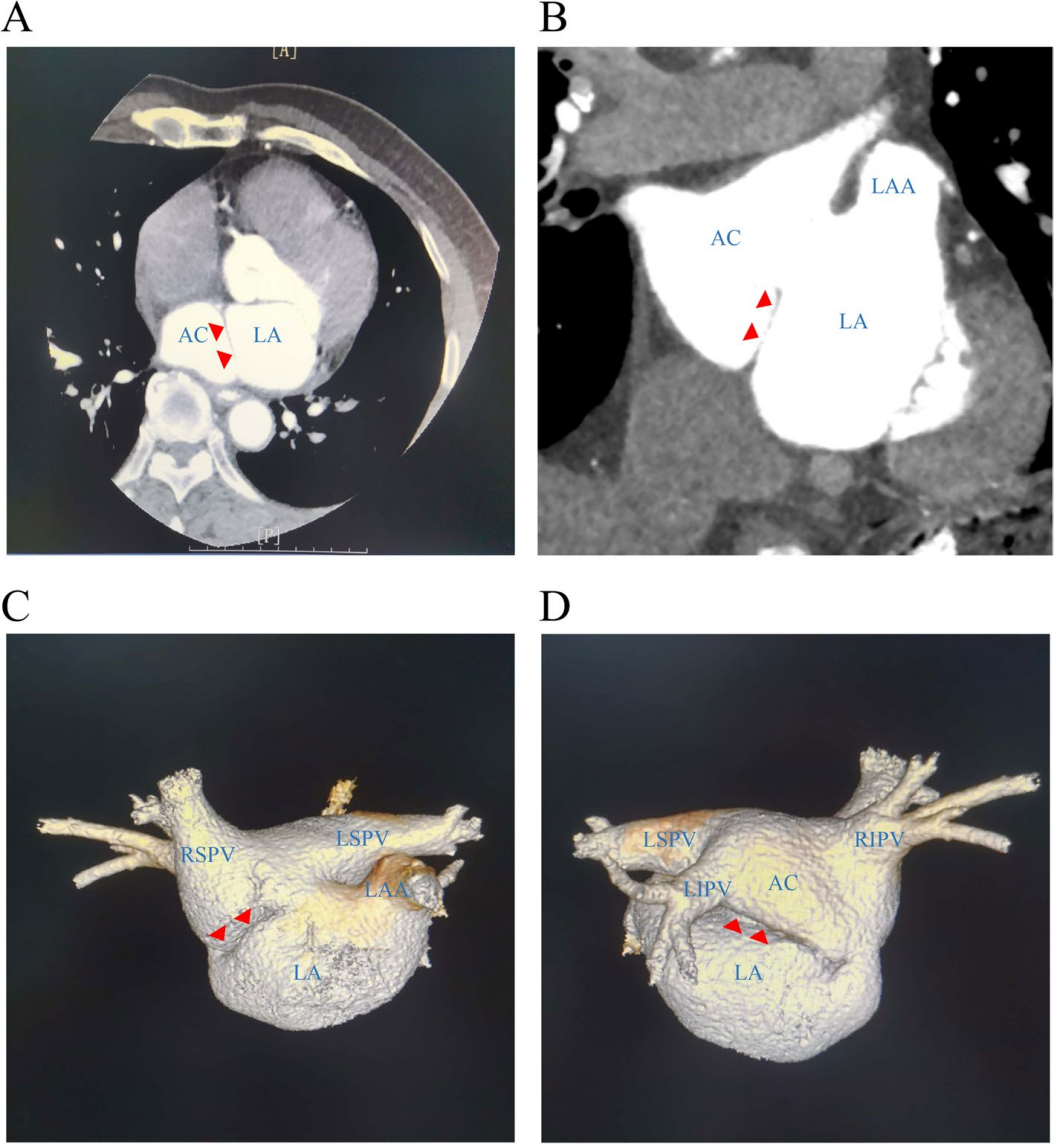
Images obtained using cardiac CTA. **A** Transverse view of the heart, red solid triangular arrowheads indicate the membrane dividing the left atrium into the accessory and true left atrial chambers. **B** Coronal view of the heart, red solid triangular arrowheads indicate the membrane dividing the left atrium into the accessory and true left atrial chambers. **C**, **D** Three-dimensional reconstruction of the left atrium by CTA. Red solid triangular arrowheads indicate the membrane. Four pulmonary veins drain into the accessory chamber and the true left atrial chamber contains the left atrial appendage (LAA) and mitral annulus. Mem: membrane; AC: accessory chamber; CTA: computed tomography angiography, LA: true left atrial chamber; LAA: left atrial appendage; LSPV: left superior pulmonary vein; LIPV: left inferior pulmonary vein; RSPV: right superior pulmonary vein; RIPV: right superior pulmonary vein

**Fig. 3:**
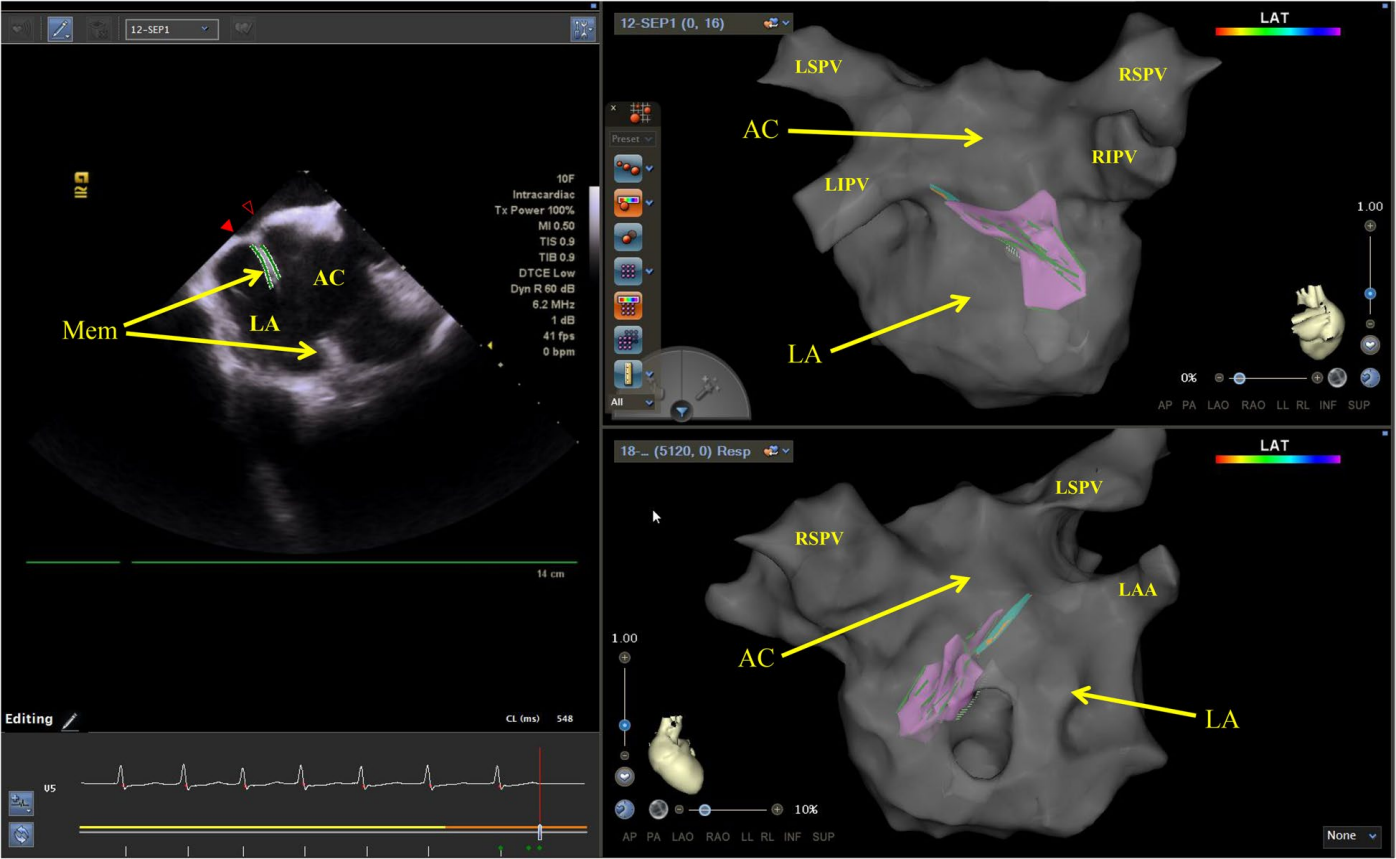
Three-dimensional intracardiac ultrasound modeling image. The pink color indicates the membrane, red hollow triangles indicate the septum entering the accessory chamber, and red solid triangles indicate the septum entering the true left atrial chamber. Mem: membrane; AC: accessory chamber; LA: true left atrial chamber; LAA: left atrial appendage; LSPV: left superior pulmonary vein; LIPV: left inferior pulmonary vein; RSPV: right superior pulmonary vein; RIPV: right superior pulmonary vein

In the preoperative cardiac computed tomography angiography (CTA), the accessory left atrial chamber was observed to receive drainage from all pulmonary veins (PVs), while the true left atrial chamber enclosed the left atrial appendage (LAA) and mitral annulus (Fig. 2B). A distinct membranous structure was also identified in the left atrium, further delineating the cor triatriatum sinister (CTS) anomaly (Fig. 2B). A three-dimensional reconstruction of the left atrium showed four PVs draining into the paraventriculus and the true atrium containing the left atrial (Fig. 2C, D). The preoperative preparation was thorough, and metoprolol tartrate tablets were discontinued approximately five half-lives prior to the procedure. Continuation of uninterrupted oral rivaroxaban was advised. A detailed model of the left atrial and membranous structure was constructed using ICE (SoundStar; Biosense Webster, Diamond Bar, CA, USA) guidance, as illustrated in Fig. 3.

**Fig. 4:**
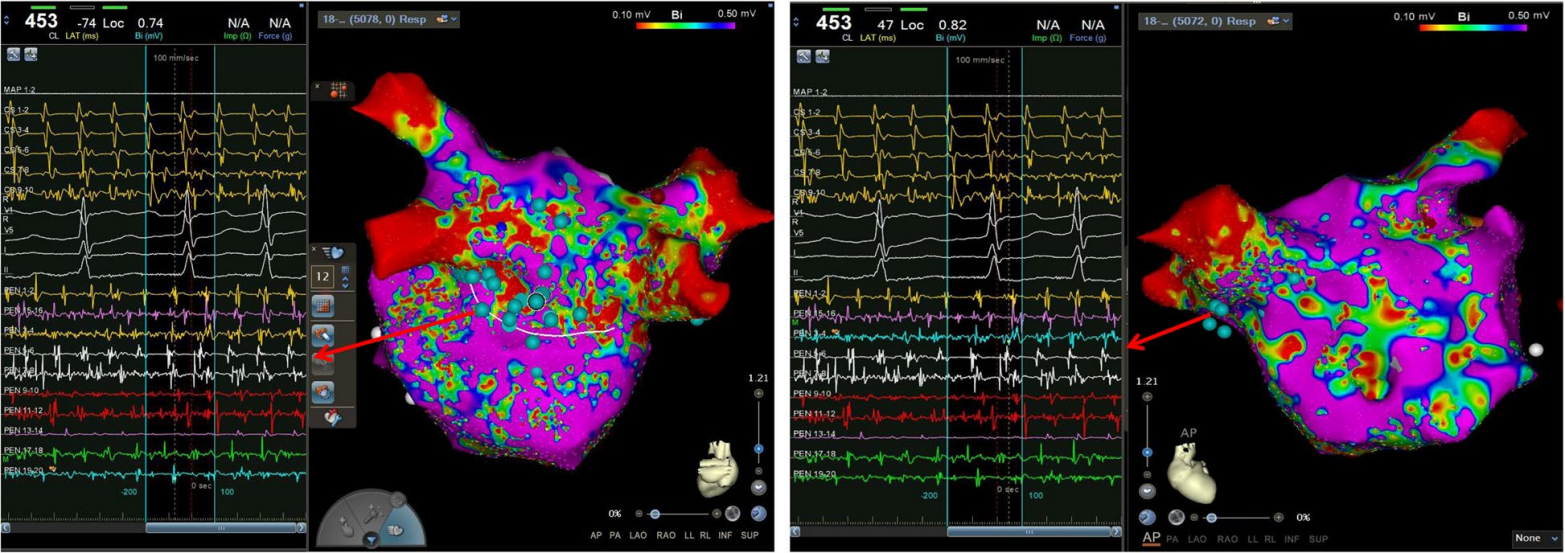
Complex fractionated atrial electrograms on mapping. The mapping revealed complex fractionated atrial electrograms, predominantly recorded in the pulmonary vein vestibule and near the bottom of the accessory left atrial chamber, specifically adjacent to the membranous structure

**Fig. 5:**
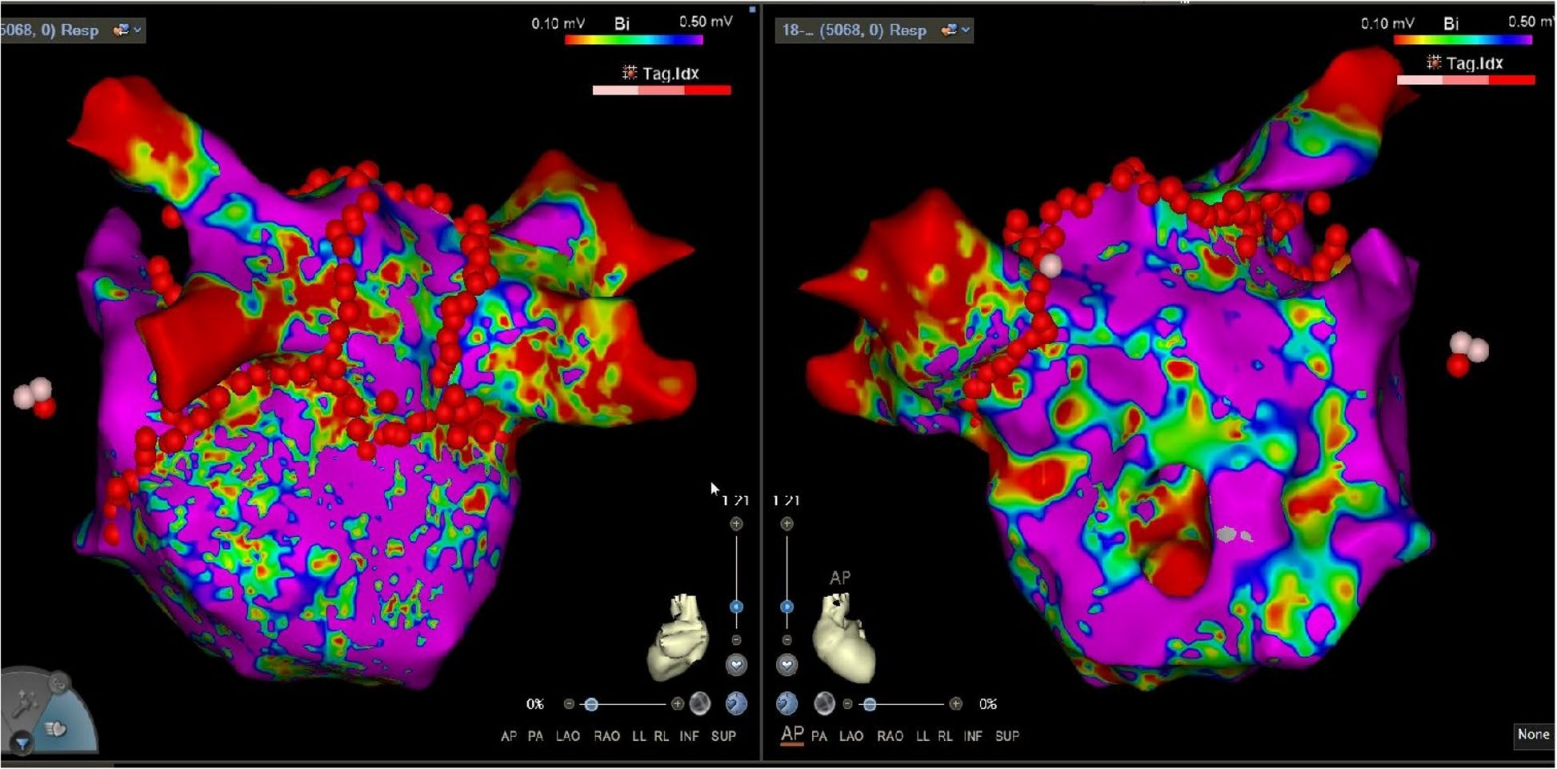
Results from the high-density substrate mapping results. Leveraging the results from the high-density substrate mapping results, an integrated approach of wide-area circumferential ablation and a small box-lesion isolation was undertaken on the posterior wall of the accessory left atrial chamber

This model facilitated the precise guidance of two atrial septal punctures, enabling the effective delivery of a catheter to the accessory left atrial chamber guided by ICE, and pulmonary venography confirmed that the catheter sheath entered the accessory chamber (Supplementary 1). Further refinement of the left atrial model was achieved using a PentaRay electrode in conjunction with the CARTO3® V7 system (Biosense Webster). The mapping revealed complex fractionated atrial electrograms (CFAEs), predominantly recorded in the PV vestibule and near the bottom of the accessory left atrial chamber, specifically adjacent to the membranous structure (Fig. 4). This detailed mapping provided valuable insights into the cardiac structure and electrical activity, crucial for the planning and execution of subsequent procedures. Leveraging the results from the high-density substrate mapping results, an integrated approach using wide-area circumferential ablation and a small box-lesion isolation was undertaken on the posterior wall of the accessory left atrial chamber (Fig. 5). This procedure was facilitated by an STSF catheter (Biosense Webster), which was coupled with the VIZIGO sheath (Biosense Webster) to provide necessary adaptability and precision.

**Fig. 6:**
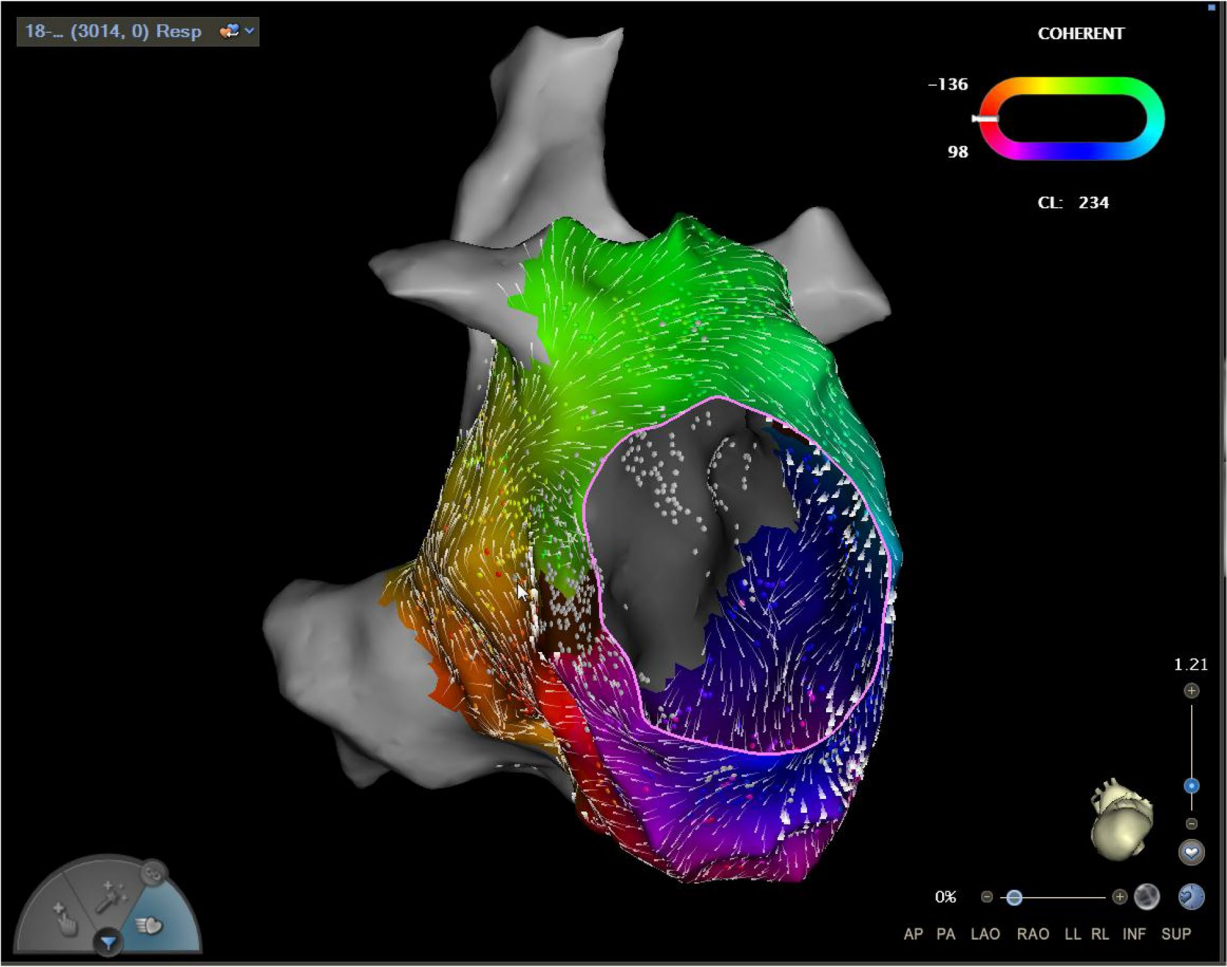
Atypical mitral isthmus-dependent left atrial flutter. Atypical mitral isthmus-dependent left atrial flutter with a tachycardia cycle length of 234 ms was characterized by an early atrial wave in the distal part of the coronary sinus

During the PV antrum and small box-lesion ablation (settings on the radiofrequency generator: power 45 W, and flow rate 15 mL/min for the PV antrum ablation; power 40 W, and flow rate 15 mL/min for the small box-lesion ablation; ablation index [AI]: 500 at the posterior wall, 450 at the superior/inferior walls and roof/bottom lines, 350–400 at the posterior wall), a notable shift in cardiac rhythm was observed. The initial AF turned into atypical mitral isthmus-dependent left atrial flutter with a tachycardia cycle length of 234 ms, characterized by an early atrial wave in the distal part of the coronary sinus, a unique pattern depicted in Fig. 6.

**Fig. 7:**
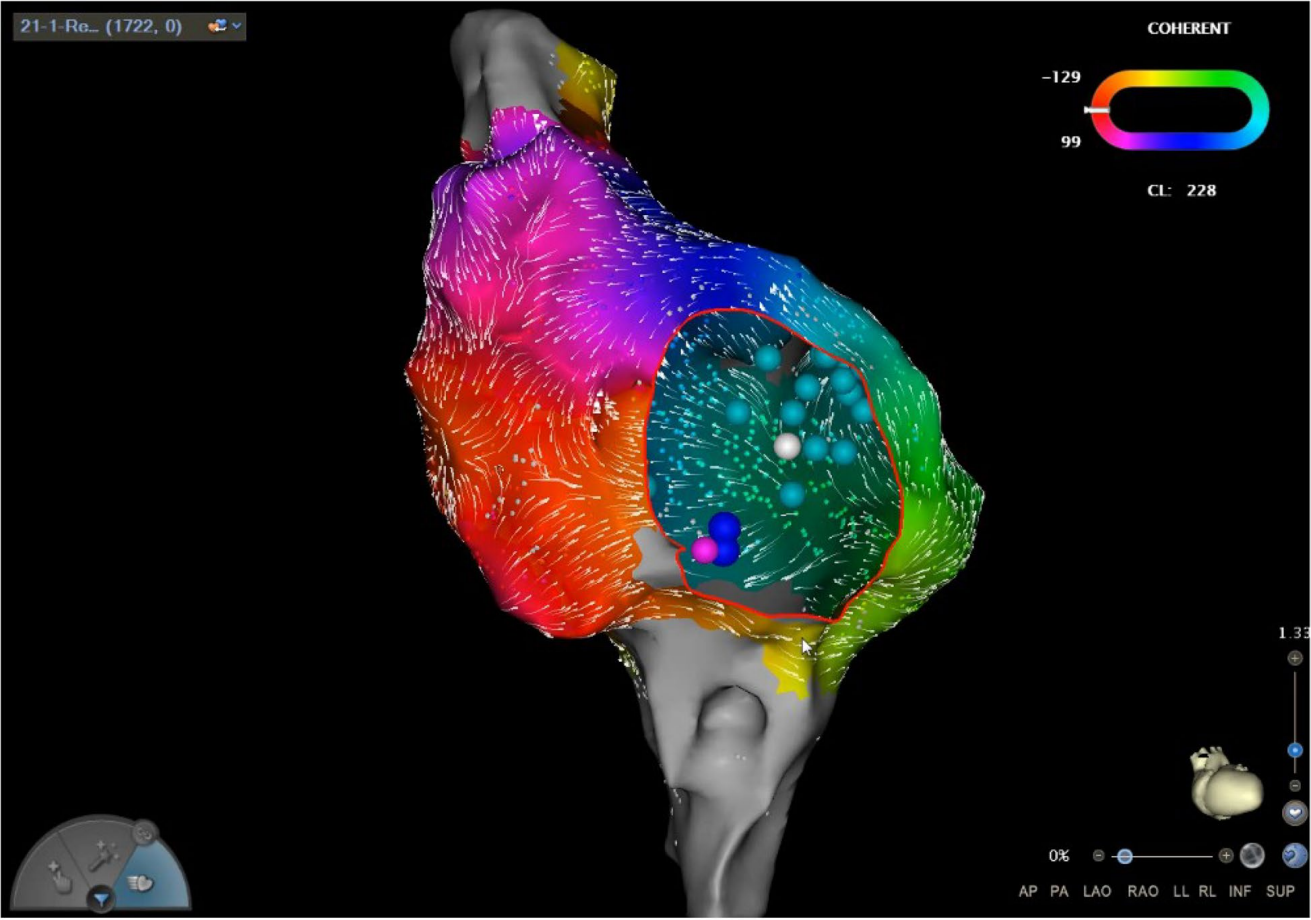
Cavo-tricuspid isthmus-dependent right atrial flutter. The cavo-tricuspid isthmus-dependent right atrial flutter with a tachycardia cycle length of 228 ms as evidenced by an early atrial wave in the proximal coronary sinus

Despite the application of a comprehensive strategy featuring both endocardial and epicardial ablation of the mitral isthmus lines (settings on the radiofrequency generator: power 45 W, and flow rate 15 mL/min for endocardial ablation of the mitral isthmus lines; power 25 W, flow rate 15 mL/min in the distal coronary vein; AI: 500–550 at the mitral isthmus lines, 400 at the distal coronary vein), the persistence of atypical mitral isthmus-dependent left atrial flutter highlighted its resistance to traditional treatment. Consequently, the pattern and perimeter of atrial flutter were noticeably altered after the adoption of vein of Marshall ethanol infusion, which is a widely-used technique for rhythm control. The condition converted to a cavo-tricuspid isthmus-dependent right atrial flutter with a tachycardia cycle length of 228 ms, evidenced by an early atrial wave in the proximal coronary sinus, as depicted in Fig. 7.

Significantly, the linear ablation at the cavo-tricuspid isthmus effectively terminated the cavo-tricuspid isthmus-dependent right atrial flutter (settings on the radiofrequency generator: power 40 W, and flow rate 15 mL/min; AI: 450). Postoperative assessments confirmed the isolation status of the bilateral PVs, the posterior wall of the accessory left atrial chamber, along with bidirectional blockage of both the mitral and cavo-tricuspid isthmus lines (Supplementary 2). ICE surveillance ascertained no pericardial effusion, stable blood pressure, and the absence of surgical complications. The patient was prescribed rabeprazole for at least one month, and rivaroxaban and amiodarone for two months post-operation. Follow-up over six months revealed sustained sinus rhythm and good overall health, and no atrial tachyarrhythmias have recurred.

## Discussion and conclusions

Cor triatriatum is an uncommon congenital cardiac anomaly, comprising 0.1–0.4% of congenital heart diseases. This condition is identified by the unique feature of the left or right atrium being split into two separate chambers due to the presence of an abnormal fibromuscular membrane [1, 2]. The more common left-sided variant, CTS often coexists with other congenital heart defects such as atrial septal defects, patent foramen ovale, or left-sided superior vena cava [2]. Diagnosis of CTS in adults is infrequent, with the symptoms depending on the degree of hemodynamic obstruction between the two left atrial chambers. As a consequence of this, adult patients with CTS and large non-obstructive fenestrations in the membrane may present as completely asymptomatic, whereas patients with small obstructive fenestrations may mimic the clinical picture of mitral stenosis due to the obstruction of flow through the membrane [3].

AF is commonly the initial symptom inpatients with CTS, occurring in approximately 32% of the cases [4]. A recent study highlighted the advantages of rhythm control therapy in patients diagnosed with AF within the first year [5]. Given our patient’s preference against surgical intervention, catheter ablation was chosen as the therapeutic approach.

The complexity of performing AF catheter ablation, particularly in cases of persistent AF, increases in patients with CTS due to the unique structural challenges of the abnormality. To navigate these challenges, our team performed comprehensive assessments using TTE and TEE along with cardiac CTA, before treatment initiation. The intricate anatomy of CTS was further clarified during the procedure via ICE, enabling precise modeling of the left atrium, the membranous structure, and its communications. Evidence from prior research underscored the vital role of ICE in enhancing AF catheter ablation [6]. This case reaffirms its significance, particularly for AF ablation when congenital left atrial abnormalities are present. ICE played a crucial role not just in guiding the catheter into the left atrium and providing a clear view of the atrial anatomy, but also in real-time monitoring of the ablation catheter’s tissue contact. This capability is essential for ensuring proper tissue contact of the catheter, thereby enhancing the safety and effectiveness of the procedure.

In previous studies, the interatrial septum was typically punctured under traditional X-ray guidance without the assistance of ICE, admitting the catheter into the true left atrial chamber. Because the transseptal catheter entered the true left atrial chamber below the membrane, isolation of all four PVs required crossing the membrane inferiorly before the catheter could be doubled back on itself to engage those structures. Such a technique was noted to complicate the maneuver significantly [7]. In contrast, our team leveraged ICE to distinctly delineate the interatrial septum and membrane relationship.

This allowed for precise catheter delivery to any chamber. We strategically chose the puncture point on the interatrial septum to enter the accessory left atrial chamber, markedly enhancing the catheter’s maneuverability. Additionally, the complexity of catheter manipulation was further reduced with the aid of the VIZIGO sheath, demonstrating a significant improvement in procedural efficiency and safety.

The catheter ablation of persistent AF often necessitates supplementary procedures beyond PV isolation, such as linear ablation, to enhance procedural efficacy. In CTS, challenges associated with the conventional mitral isthmus line ablation pathway have been noted. A notable alternative pathway, as suggested in literature, involves ablation from the anterior wall of the mitral annulus to the left superior PV, requiring targeted ablations on each side of the membrane. However, the potential heightened risk of delayed conduction in the LAA resulting from this alternative pathway remains unexplored in existing literature [8]. In addressing atypical mitral isthmus-dependent left atrial flutter in CTS, our team innovatively applied vein of Marshall ethanol infusion to achieve effective mitral isthmus blockage. This approach circumvents the impact of the membrane on catheterization.

Karimianpour et al. discovered a significant correlation in patients exhibiting atrial flutter within the context of CTS. Their investigation revealed CFAEs in the membrane, characterized by a latent entrainment and a proximity of the post-pacing interval to the tachycardia cycle length. This observation implies that the membrane may constitute an arrhythmogenic substrate [9]. Similarly, in the present case, CFAEs were identified near the base of the accessory left atrial chamber, particularly adjacent to the membranous structure, suggesting a potential involvement of the membrane in the genesis of AF within CTS.

In conclusion, an in-depth comprehension of the anatomical nuances is essential for successful radiofrequency ablation in patients with AF and CTS. Preoperative assessment of cardiac structure involves TTE and TEE, as well as cardiac CTA. During the procedure, ICE facilitates detailed modeling of the left atrium, including the membranous structure and its openings, thus providing a clearer understanding of CTS. The utilization of the VIZIGO sheath facilitates catheter manipulation, thereby mitigating operational challenges. It is noteworthy that the membrane within the CTS may serve as a potential substrate for arrhythmias and warrants further validation through larger sample studies.

### Supplementary Information

Supplementary Material 1.

Supplementary Material 2.

## Data Availability

No datasets were generated or analysed during the current study.

## Abbreviations

CTS: Cor triatriatum sinister
AF: Atrial fibrillation
CFAEs: Complex fractionated atrial electrograms
ICE: Intracardiac echocardiography
TTE: Transthoracic echocardiography
TEE: Transesophageal echocardiography
CTA: Computer tomography angiography
PVs: Pulmonary veins
AI: Ablation index
Mem: Membrane
AC: Accessory chamber
LA: True left atrial chamber
LAA: Left atrial appendage
LSPV: Left superior pulmonary vein
LIPV: Left inferior pulmonary vein
RSPV: Right superior pulmonary vein
RIPV: Right superior pulmonary vein
