# Supplementary material for: Exploring new frontiers: a rare case of catheter ablation for persistent atrial fibrillation in a patient with cor triatriatum sinister guided by intracardiac echocardiography
Source: J Cardiothorac Surg. 2024 Jun 22;19:355. doi: 10.1186/s13019-024-02859-9 (PMC11193257; doi:10.1186/s13019-024-02859-9)
Supplement: Supplementary file 1 — Supplementary Material 1. [file 13019_2024_2859_MOESM1_ESM.pptx]

## Slide 1
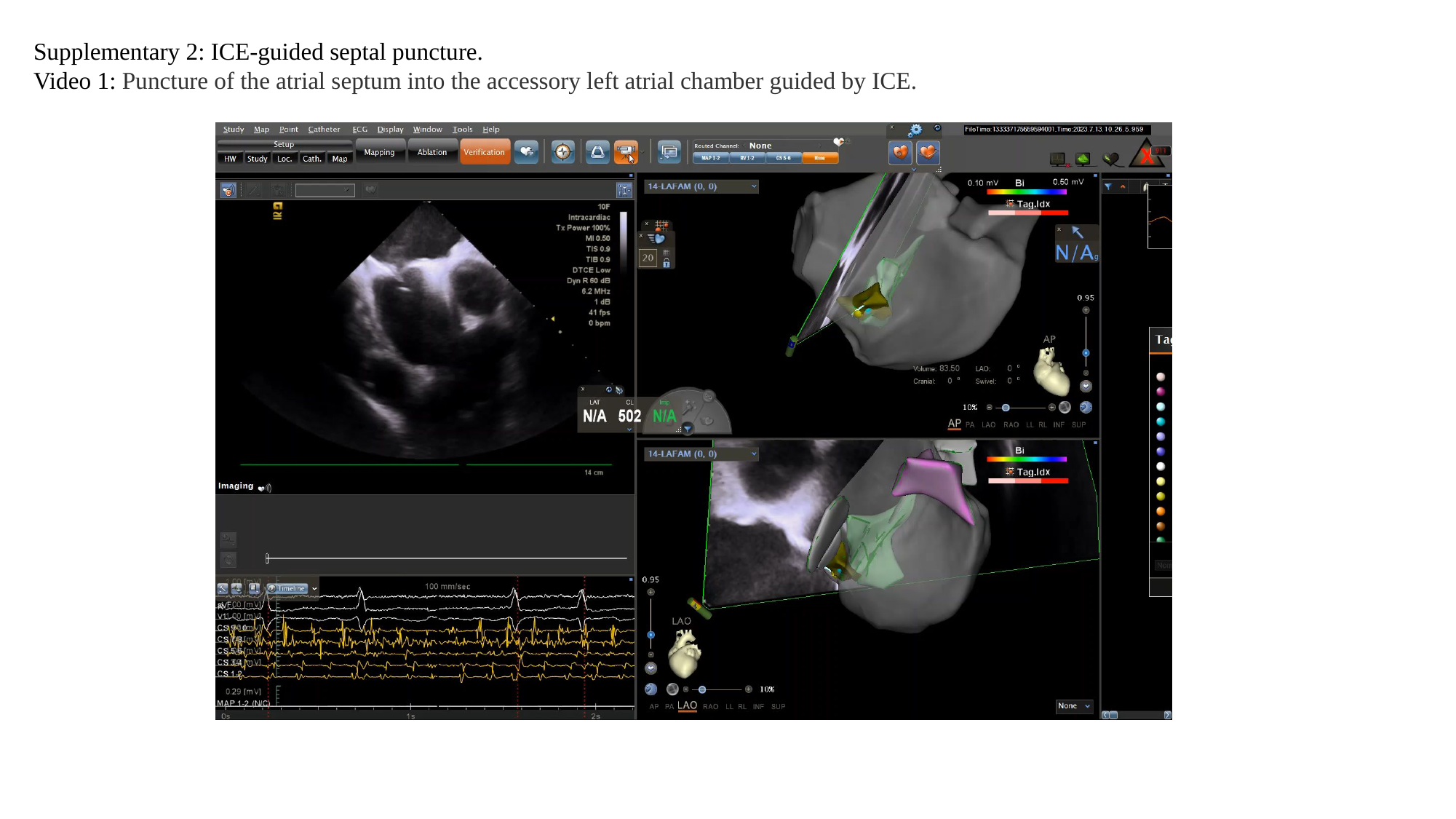

Supplementary 2: ICE-guided septal puncture.
Video 1: Puncture of the atrial septum into the accessory left atrial chamber guided by ICE.

## Slide 2
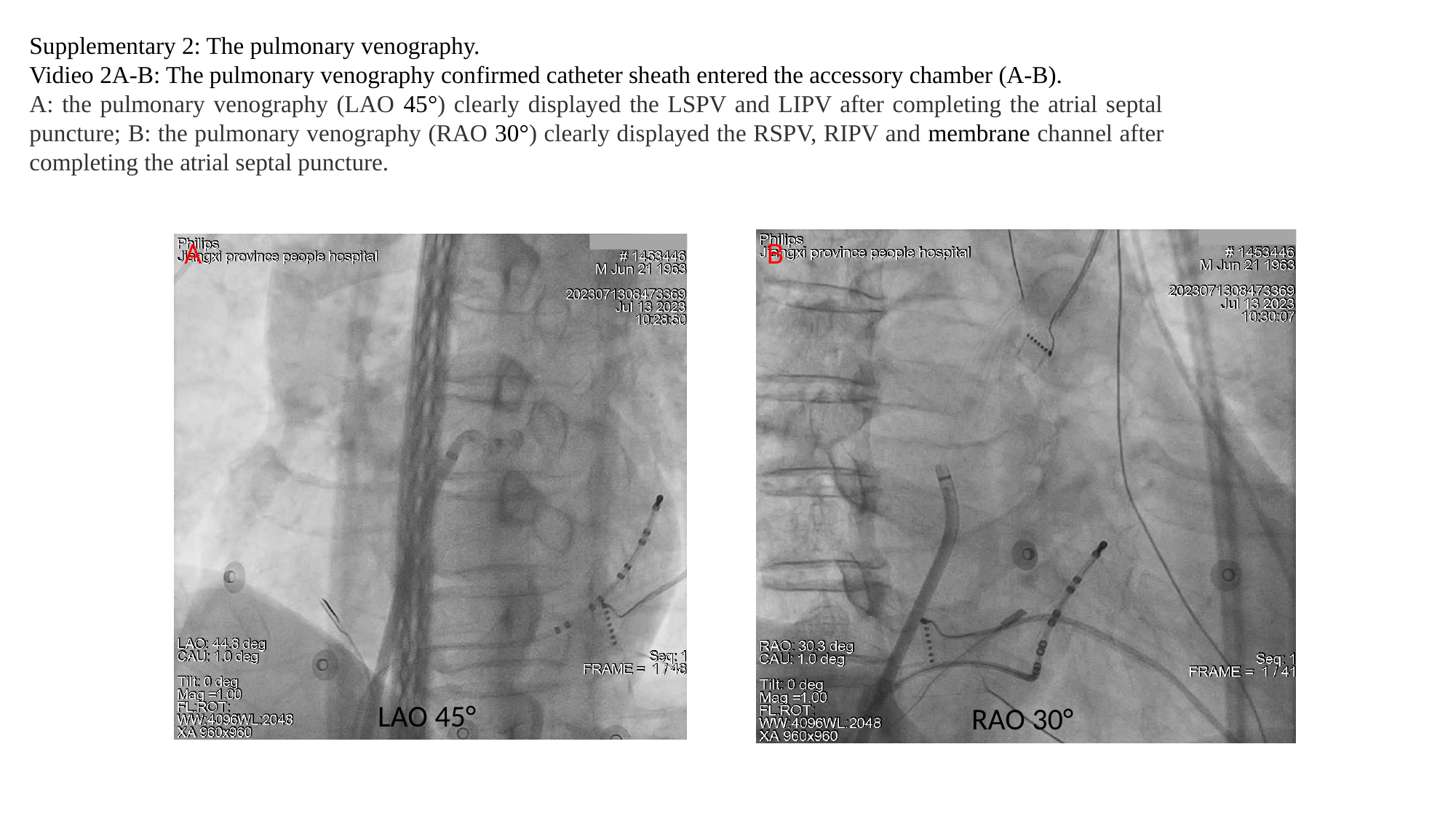

Supplementary 2: The pulmonary venography.
Vidieo 2A-B: The pulmonary venography confirmed catheter sheath entered the accessory chamber (A-B).
A: the pulmonary venography (LAO 45°) clearly displayed the LSPV and LIPV after completing the atrial septal puncture; B: the pulmonary venography (RAO 30°) clearly displayed the RSPV, RIPV and membrane channel after completing the atrial septal puncture.
A
B
LAO 45°
RAO 30°
