# Supplementary material for: Exploring new frontiers: a rare case of catheter ablation for persistent atrial fibrillation in a patient with cor triatriatum sinister guided by intracardiac echocardiography
Source: J Cardiothorac Surg. 2024 Jun 22;19:355. doi: 10.1186/s13019-024-02859-9 (PMC11193257; doi:10.1186/s13019-024-02859-9)
Supplement: Supplementary file 2 — Supplementary Material 2. [file 13019_2024_2859_MOESM2_ESM.docx]

**Supplementary 2: Detailed description: Postoperative assessments confirmed the isolation status of the bilateral PVs, the posterior wall of the accessory left atrial chamber, along with bidirectional blockage of both the mitral and cavo-tricuspid isthmus lines.**

No potentials were recorded in the pulmonary veins and the small box lesion to verify the block. The absence of potentials in the small box lesion suggests block in the roof lines and the bottom lines, while dual-site pacing from different locations on both sides was used to verify bidirectional block in both the mitral and cavo-tricuspid isthmus lines.

1) Validation of pulmonary veins block (Figure 1A-D).


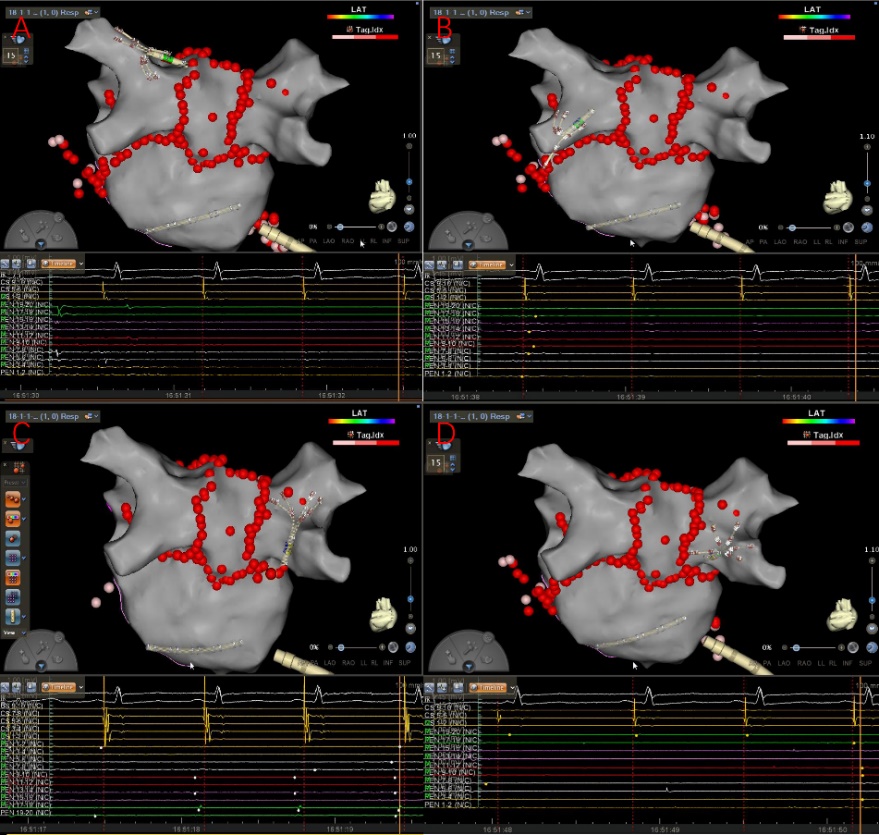


Figure 1: Validation of the pulmonary veins block. No potentials were identified in (A) the LSPV, (B) the LIPV, (C) the RSPV or (D) the RIPV. LSPV: left superior pulmonary vein; LIPV: left inferior pulmonary vein; RSPV: right superior pulmonary vein; RIPV: right superior pulmonary vein.

2) Validation of block in the small box lesion, the roof lines and the bottom lines (Figure 2A-B).


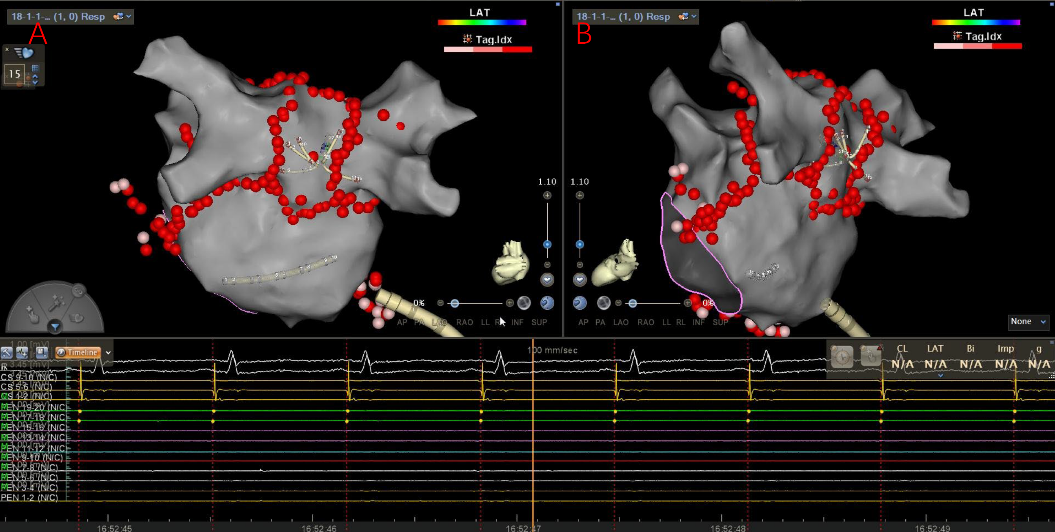


Figure 2: Validation of the block in the small box lesion, the roof lines and the bottom lines. No potentials were identified in the small box lesion (A and B), and the absence of potentials in the small box lesion also suggests a block in the roof lines and the bottom lines.

3) Validation of bidirectional block in the mitral isthmus lines (Figure 3A-B).


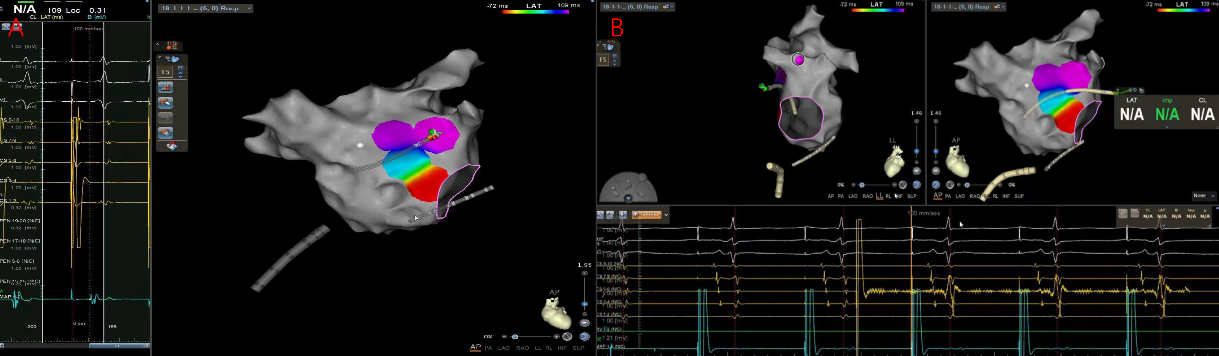


Figure 3: Validation of the bidirectional block in the mitral isthmus lines. A. A bottom-to-top (red-to-purple) activation sequence in the anterior wall was interpreted as indicative of unidirectional conduction block in the mitral isthmus lines during pacing from mid-coronary sinus with a recording site just inferior to the ablation lines. B. A proximal-to-distal activation sequence in coronary sinus electrode was interpreted as indicative of other unidirectional conduction block in the mitral isthmus lines during left atrial appendage pacing.

4) Validation of bidirectional block in the cavo-tricuspid isthmus lines (Figure 4A-C).


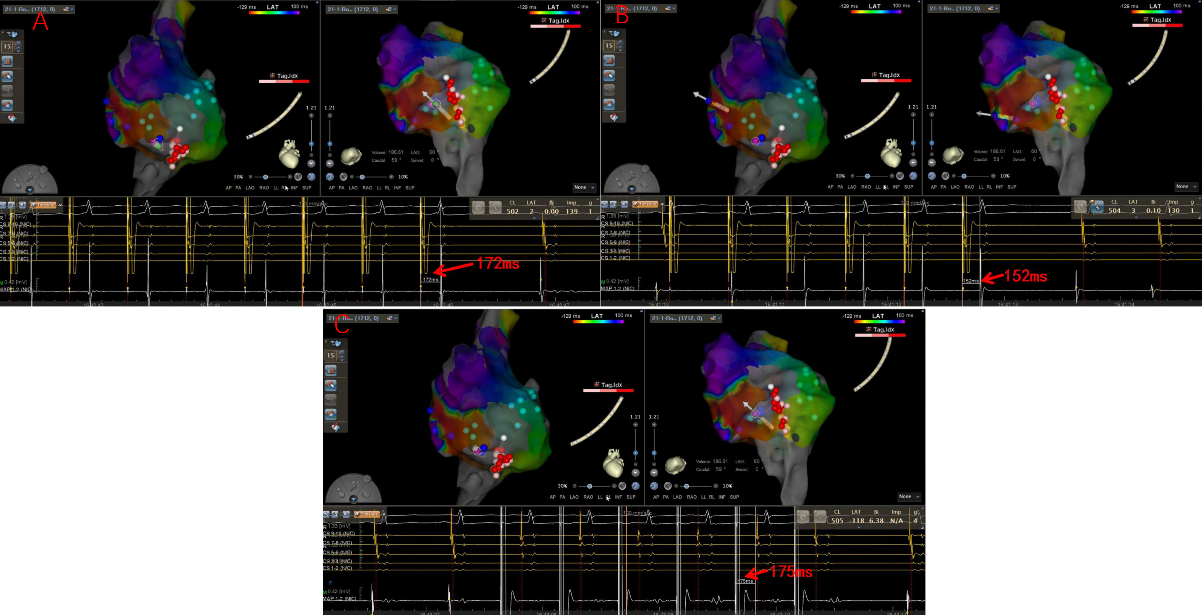


Figure 4: Validation of the bidirectional block in the cavo-tricuspid isthmus lines. Panels A and B indicate a unidirectional conduction block in the cavo-tricuspid isthmus lines, meanwhile, panel C indicates another unidirectional conduction block in the cavo-tricuspid isthmus lines. A. SA interval =172 ms during proximal coronary sinus pacing. B. SA interval=152 ms during proximal coronary sinus pacing. C. SA interval=175 ms during pacing at the distal end of the ablation catheter. SA: stimulus atrial.
